# Cold-Shock Domains—Abundance, Structure, Properties, and Nucleic-Acid Binding

**DOI:** 10.3390/cancers13020190

**Published:** 2021-01-07

**Authors:** Udo Heinemann, Yvette Roske

**Affiliations:** Crystallography, Max Delbrück Center for Molecular Medicine, 13125 Berlin, Germany; yroske@mdc-berlin.de

**Keywords:** cold-shock domain, cold-shock protein, RNA-binding domain, nucleic-acid binding, gene regulation, OB fold, Y-box binding protein, domain fold, protein structure, protein stability and folding

## Abstract

**Simple Summary:**

Proteins are composed of compact domains, often of known three-dimensional structure, and natively unstructured polypeptide regions. The abundant cold-shock domain is among the set of canonical nucleic acid-binding domains and conserved from bacteria to man. Proteins containing cold-shock domains serve a large variety of biological functions, which are mostly linked to DNA or RNA binding. These functions include the regulation of transcription, RNA splicing, translation, stability and sequestration. Cold-shock domains have a simple architecture with a conserved surface ideally suited to bind single-stranded nucleic acids. Because the binding is mostly by non-specific molecular interactions which do not involve the sugar-phosphate backbone, cold-shock domains are not strictly sequence-specific and do not discriminate reliably between DNA and RNA. Many, but not all functions of cold shock-domain proteins in health and disease can be understood based of the physical and structural properties of their cold-shock domains.

**Abstract:**

The cold-shock domain has a deceptively simple architecture but supports a complex biology. It is conserved from bacteria to man and has representatives in all kingdoms of life. Bacterial cold-shock proteins consist of a single cold-shock domain and some, but not all are induced by cold shock. Cold-shock domains in human proteins are often associated with natively unfolded protein segments and more rarely with other folded domains. Cold-shock proteins and domains share a five-stranded all-antiparallel β-barrel structure and a conserved surface that binds single-stranded nucleic acids, predominantly by stacking interactions between nucleobases and aromatic protein sidechains. This conserved binding mode explains the cold-shock domains’ ability to associate with both DNA and RNA strands and their limited sequence selectivity. The promiscuous DNA and RNA binding provides a rationale for the ability of cold-shock domain-containing proteins to function in transcription regulation and DNA-damage repair as well as in regulating splicing, translation, mRNA stability and RNA sequestration.

## 1. Introduction

Proteins are made of compact domains with defined three-dimensional folding and of natively unstructured polypeptide segments. These globular domains are recurrent structural elements, serving as parts sets of molecular evolution and often appearing in multiple proteins that may or may not share common biochemical or biological functions. The number of domain folds is limited; an early hypothesis speculated about the presence of “one thousand families for the molecular biologist” [1]. As with domain folds in the entire protein universe, there is a limited repertoire of RNA-binding domains (RBDs) [2] including the cold-shock domain (CSD). Canonical RBDs preferentially bind short single-stranded sequence motifs in RNA, but binding to structured regions of RNA is also observed [3].

The total number of human RNA-binding proteins (RBPs) is estimated at more than 800 in both proliferating HeLa cells [4] and in an embryonic kidney cell line [5]. An analysis of the RNA interactome to the sub-domain level revealed more than 1100 RNA-binding sites in more than 500 RBPs including CSD-containing proteins [6].

A recent review of CSD-containing proteins [7] directed its focus on their domain structure and sequence motifs in interacting nucleic acids. Here, we compare bacterial cold-shock protein (CSPs) with CSDs occurring in human or other eukaryotic proteins. We focus on the common structural, biophysical and nucleic acid-binding properties of these evolutionarily related domains which share a conserved geometry of binding single-stranded DNA or RNA (ssDNA or ssRNA) and limited sequence or nucleic acid-type selectivity. Some of these common principles were revealed in very recent structural studies [8,9,10,11,12,13,14].

## 2. Definition, Abundance and Discovery of Cold-Shock Domains

### 2.1. Definition and Basic Properties of Cold-Shock Domains

Almost three decades ago, a common oligonucleotide/oligosaccharide-binding (OB) fold was identified in four proteins unrelated by sequence: staphylococcal nuclease, the anticodon-binding domain of aspartyl-tRNA synthetase, and the β-subunits of two *Escherichia coli* toxins, heat-labile enterotoxin and verotoxin-1. The OB fold as present in these proteins is characterized by 70–150 amino-acid (aa) residues organized into a five-stranded all-antiparallel β-barrel which is frequently capped by an α-helix located between strands β3 and β4. Oligonucleotide and oligosaccharide ligands bind to a conserved surface area on the barrel [15]. There is little to no sequence conservation between OB-fold proteins, but ssDNA and ssRNA bind with conserved polarity to oligonucleotide-binding family members [16].

OB-fold proteins including staphylococcal nuclease, the bacterial enterotoxins and the MOP (molybdate/tungstate binding)- and TIMP (tissue inhibitor of metalloproteases)-like proteins have been classified into superfamilies. The nucleic acid-binding proteins constitute the largest OB-fold superfamily, including protein families such as the single-strand DNA-binding (SSB) proteins and RNA-binding RNB domain-like proteins. Among the nucleic acid-binding proteins, the cold-shock DNA-binding domain-like proteins constitute the largest family. This protein family can be further divided into proteins containing (proper) cold-shock domains, which are the subject of this review, and other domains including the S1, IF2-type S1, S12, S17, and S28e domains first identified in proteins of the small ribosomal subunit [17]. The taxonomic distribution of the abundant S1 domain-containing family of OB-fold proteins was recently reviewed [18]. Frequently, multiple OB domains are present in oligomeric proteins or within one polypeptide chain. The human CTC1-STN1-TEN1 (CST) complex essential for telomere maintenance, may serve as an impressive example. Here, all subunits contain OB-fold domains two of which (OB-F and OB-G of CTC1) are involved in ssDNA binding as demonstrated by cryo-electron microscopy (cryo-EM) [19].

**Figure 1:**
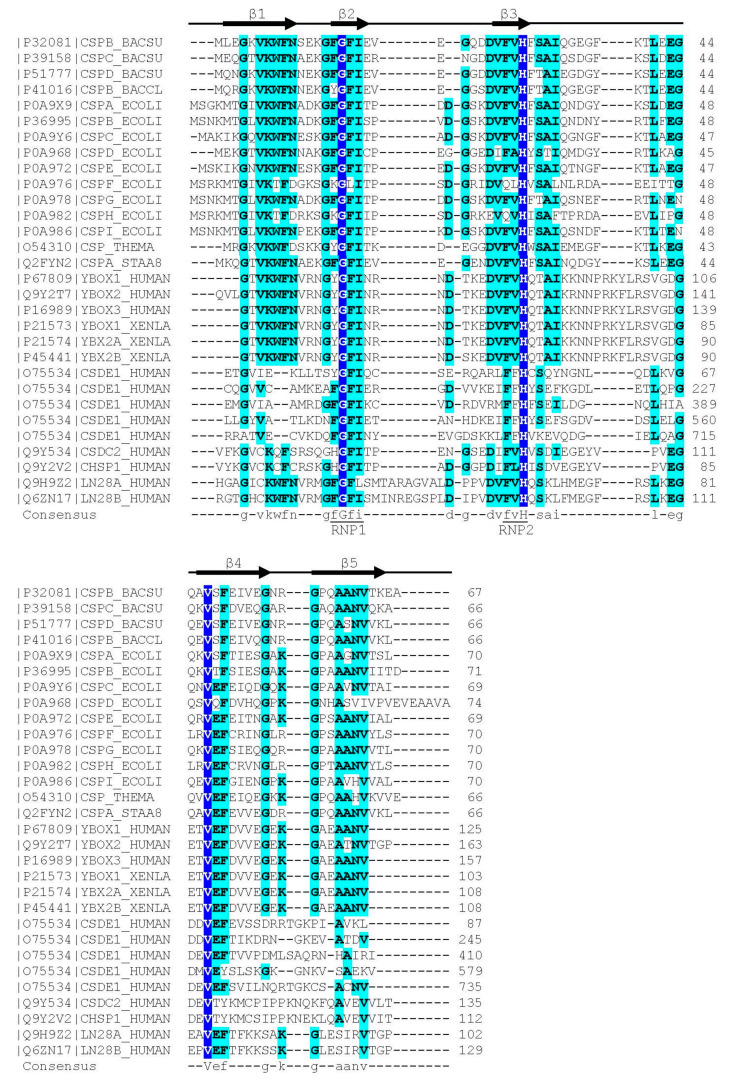
Sequence alignment of representative bacterial CSPs and CSDs from human proteins. The *Xenopus laevis* FRGY1 and FRGY2 (YBOX1, YBX2A, YBX2B) proteins are also included. Human CSDE1 contains five CSDs, all other proteins contain or consist of a single CSD. Proteins are identified by their Uniprot [22] entry number and name. The secondary-structure annotation atop the sequence follows *Bs*CspB, the first CSP for which a crystal structure was determined [23]. Residues conserved across all aligned CSDs are highlighted on dark blue background and shown with capital letters in the consensus sequence. Residues conserved in ≥50% of the sequences are shown on a light blue background and with lower-case letters in the consensus. Sequences were aligned using the Clustal Omega server [24]. The sequence motifs RNP1 ([YF]-G-F-I) and RNP2 ([YF]-[YF]-H) are associated with RNA binding and indicated according to Prosite [25].

In contrast to the wider group of OB-fold proteins, the bacterial CSPs and eukaryotic CSDs are of very similar length of about 70 aa and share clearly conserved sequences. The presence of RNP1 and RNP2 sequence motifs [20] provided an early hint towards a function of CSDs in binding single-stranded nucleic acids. As in other RBPs, the RNP motifs [21] are placed within β-strands. These RNP motifs contain some of the most highly conserved residues in the set of representative bacterial CSPs and eukaryotic CSDs displayed in Figure 1. In general, sequence conservation in CSPs and CSDs is higher in β-strands than in loop regions. Whereas bacterial CSPs are small proteins consisting, as a rule, of a single CSD only, their eukaryotic homologs are proteins of variable length and domain composition.

### 2.2. Abundance of Cold-Shock Domains

Analysis of the SCOP database [26] finds 18 protein superfamilies within the OB fold including a nucleic acid-binding (NAB) superfamily. The 17 protein families within the NAB include the cold-shock DNA-binding protein (CSDB) family, which further separates into 32 domain types of which the CSD is one. The SMART database [27] lists the CSD under accession number SM00357. As of 01 Dec 2020, SMART contained 80,336 CSDs in 70,472 proteins. Of these proteins, 90.9% occurred in bacteria and 7.3% in eukaryotes. SMART lists 29 CSDs in the human proteome.

**Figure 2:**
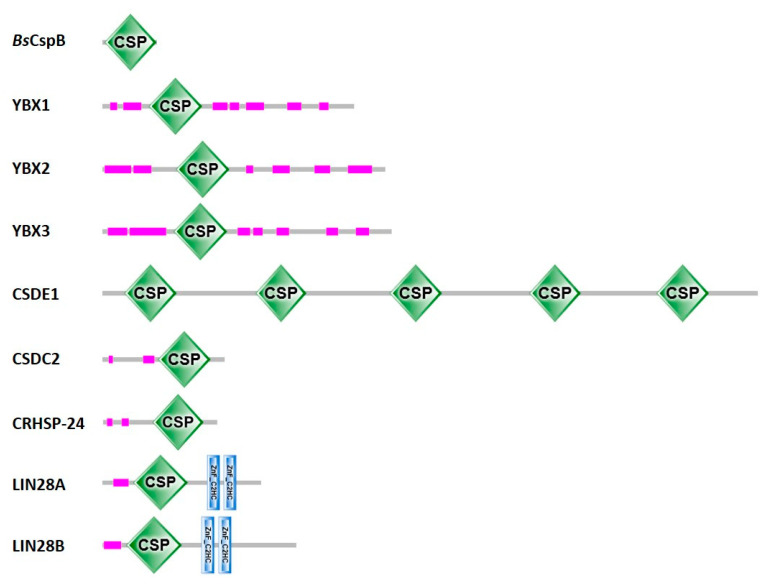
Proteins with cold-shock domains. Domain annotations for one representative bacterial CSP and human CSD-containing proteins according to SMART [27]. For CSDE1, Pfam [31] agrees with the domain annotation shown here. Uniprot [22] annotates two additional CSDs in CSDE1, one between CSD3 and CSD4 and one between CSD4 and CSD5, as well as two additional truncated CSDs, one between CSD1 and CSD2 and one between CSD2 and CSD3. InterPro [32] annotates a total of nine CSDs in CSDE1, those shown here and four additional CSDs filling the gaps. CSDs are displayed as green diamonds labeled “CSP”, the stunted CCHC-type zinc fingers (zinc knuckles) present in the LIN28 proteins as blue vertical bars, and low-complexity sequences as pink bars. Proteins are drawn to scale.

The large majority of CSDs are in single-domain bacterial CSPs. Gram-negative *E. coli* contains nine *csp* genes of which *cspA*, *cspB*, *cspG* and *cspI* are cold-inducible, while the others are not [28]. Gram-positive *Bacillus subtilis* contains three CSP paralogs, *Bs*CspB, *Bs*CspC and *Bs*CspD [29]. In general, the number of CSPs in different bacteria is variable and evidently unlinked to habitat or preferred growth temperature. The larger eukaryotic proteins contain between one and five (or more) CSDs, frequently in combination with natively unfolded polypeptide regions and less often with other domains of known fold [30] (see Figure 2).

### 2.3. Discovery of Cold-Shock Domains

In 1987 Jones et al. observed that a sudden down-shift in growth temperature of *E. coli* W3110 cultures caused changes in protein abundance with many proteins being down-regulated and a small number, the cold-shock proteins, being up-regulated [33]. Subsequently, a small 7.4-kDa protein named CS7.4 was discovered after down-shift of *E. coli* growth temperature from 37 °C to 10 or 15 °C. The rate of CS7.4 synthesis increased dramatically within ~1 h after temperature down-shift. The corresponding gene (*cspA*) was cloned and shown to encode a hydrophilic 70-aa polypeptide which binds to and stimulates the transcription of the CCAAT-containing promoters of the HN-S protein and of *gyrA* [34]. In this review, we shall use a notation where bacterial CSPs are identified by their source organism and gene name; hence CS7.4 will be referred to as *Ec*CspA from here on.

Expression of the *E. coli cspA* gene could be further induced by chloramphenicol at 15 °C. Whereas *cspA* up-regulation by cold shock was transient, antibiotic-stimulated gene expression was constitutive [35] indicating the presence of more than one regulatory mechanism for *cspA* expression. Furthermore, the paralogous *E. coli* genes *cspB*, *cspG* and *cspI* were cold-inducible while other paralogs were not [36], and the *E. coli* “cold-shock response” could be induced by other stimuli, such as inhibitors of translation [37]. Therefore, the cold-shock response may be seen as just one aspect of a more general stress-response scheme and the terms “cold-shock protein” or “cold-shock domain” may be regarded as misnomers; but they are here to stay. However, it remains undisputed that some proteins with CSDs may confer cold protection, as recently demonstrated for the *Ga16676* gene of the psychrophilic yeast *Glaciozyma antarctica* which encodes a protein with N-terminal CSD. Overexpression of this gene in *E. coli* was reported to induce increased bacterial cell growth at 10 °C [38].

Paralogous CSPs may have distinct or redundant function in their bacterial hosts. In *Staphylococcus aureus*, for example, only *Sa*CspA but no other CSP can stimulate the biosynthesis of the pigment staphyloxanthin (STX). However, a single amino-acid mutation (E58P) enables the paralog *Sa*CspC to restore STX production in a *cspA* deletion strain [39]. *Sa*CspA post-transcriptionally modulates target-gene expression by binding to sites in the 3′-untranslated regions (3′UTRs) of their mRNAs [40].

Temperature-regulated genes evolutionarily unrelated to the CSD have been described in eukaryotes. For example, *Saccharomyces cerevisiae* TIP1 (temperature shock-inducible protein 1) was found upregulated by both cold and heat shock [41]. Yeast NSR1 (nuclear localization sequence-binding protein-1) is another example for a protein that is up-regulated under cold shock. NSR1 does not contain a CSD and is structurally related to mammalian nucleolin [42]. In human cells, the cold-inducible RNA-binding protein CIRBP serves a well-documented role in circadian regulation [43], but its RNA association is mediated by an RNA-recognition motif (RRM) and not by a CSD [44].

The most extensively studied human protein containing a CSD is YBX1, the Y-box binding protein 1, also known as YB-1, CBF-A (CCAAT-binding transcription factor I subunit A), DBPB (DNA-binding protein B), EFI-A (enhancer factor I subunit A), or NSEP1 (nuclease-sensitive element-binding protein 1). YBX1 was identified as a basic protein specifically binding to the Y-box, a *cis*-acting element in regulating the expression of HLA class-II genes containing an inverted CCAAT box (ATTGG). An inverse correlation between YBX1 and levels of HLA DRβ chain mRNA was observed [45]. When the homology between bacterial *Ec*CspA and a domain in human YBX1, the CSD, was noted, the cold-shock response was linked to DNA binding and the conservation of the CSD across evolutionarily distant organisms established [46]. Cellular functions of YBX1 related to RNA binding were also discovered early on, for example YBX1’s ability to stabilize mRNA by association of its CSD with the 5′-cap structure and by destabilizing the cap interaction with the cap-binding complex eIF4F [47]. Recently, YBX functions related to RNA binding have received increased attention [48].

Although functions of YBX1 are generally linked to DNA and/or RNA binding, some cellular activities, e.g., in regulating cytokinesis, may be facilitated by phosphorylation-dependent protein-protein interactions [49]. Under oxidative stress, nuclear YBX1 physically interacts with the DNA-repair enzyme DNA glycosylase NEIL2 and stimulates its base-excision activity [50]. In humans, YBX1 and other CSD-containing proteins serve a plethora of biological functions [51,52,53] and are broadly involved in disease development and progression [54,55]. Functions of YBX1 in DNA-damage repair, transcription regulation, splicing and translation, and their cellular consequences in cancer are summarized in a recent review [56], and biological roles of YBX1 dependent on RNA binding, including formation of messenger ribonucleoprotein (mRNP) and mRNA stabilization were reviewed as well [48,57].

YBX1 is localized to various subcellular compartments. In addition to nuclear and cytoplasmic YBX1, a secreted form arising from a non-classical export pathway was also described [58] which may be linked to a more recently discovered function of YBX1 in regulating the sorting of small non-coding RNAs into extracellular vesicles [59]. Furthermore, the formation of cytoplasmic stress granules (SGs) by tRNA-derived stress-induced RNAs (tiRNAs) is mediated by YBX1 through direct YBX1-CSD association with the tiRNA [60]. The YBX1 CSD was shown to bind angiogenin-produced tiRNA^Ala^ to displace the cap-binding complex eIF4F from capped mRNA, inhibit translation and induce SG assembly [61]. Some tiRNAs were reported to have a tumor-suppressive role in breast-cancer cells by displacing YBX1 from the 3′UTRs of oncogenic transcripts [62].

Two human paralogs of YBX1 are known. YBX2 is also known as YB-2, DNA-binding protein C (DBPC) or CSDA3; YBX3 is also known as YB-3, DBPA, CSDA or *zonula occludens* 1 (ZO-1) associated nucleic acid-binding protein (ZONAB). YBX1, YBX2 and YBX3 share a common domain organization with an N-terminal region followed by a CSD and differently spaced alternating arginine-rich and acidic regions (Figure 2). With three type-conserved amino-acid replacements between YBX1 and YBX2 and identical sequences in YBX1 and YBX3 the CSDs of the human Y-box proteins are extremely well conserved. However, clearly different phenotypes of gene knockouts are observed in mice where the *YBX1* knockout is embryonic lethal, while *YBX2* and *YBX3* knockouts are associated with compromised offspring fertility [22].

Stress-induced YBX3 stabilizes the mRNA of p21^WAF1/CIP1^, a central element of the cellular stress response, by binding into its 3′UTR and enhances its translation. YBX3 thereby promotes cell survival under conditions of cytotoxic stress [63]. YBX3 is recruited to tight junctions by ZO-1 and mediates Rho-regulated cyclin D1 promoter activation by interacting with the Rho activator GEF-H1 [64]. YBX3 and ZO-1 cooperate in controlling the expression of ErbB-2 [65].

YBX1 homologs are present in many organisms including *Xenopus*, where FRGY1 has a broad tissue distribution while FRGY2 expression is limited to germ cells [66]. In analogy to human YBX1, FRGY1 and FRGY2 act as transcription factors binding to the CCAAT-containing Y-box of *Xenopus* hsp70 genes. In frog oocytes, certain transcripts are masked by FRGY2, leading to translational repression [67]. Four Y-box binding proteins (CEY-1 to CEY-4) are present in *Caenorhabditis elegans*. These proteins are essential for fertility and function in the formation of large polysomes [68].

Cold shock domain-containing protein E1 (CSDE1), also known as Upstream of N-Ras (UNR), contains multiple CSDs suggesting a role as multivalent nucleic acid-binding protein. The five bona fide CSDs of human CSDE1 confer high affinity for single-stranded DNA or RNA, but not for dsDNA or double-stranded and structured RNA. Both ssDNA and ssRNA are bound without distinct sequence preference, but simple homopolymers are bound with reduced affinity. CSDE1 primarily localizes to the cytoplasm where it may associate with cytoplasmic mRNA in vivo [69] and was ascribed the ability to both enhance or repress mRNA translation and both reduce or increase RNA abundance [70]. CSDE1 is highly expressed in human embryonic stem cells (hESCs) and functions to arrest them in their undifferentiated state. In addition, CSDE1 was shown to bind the mRNAs of fatty acid-binding protein 7 (FABP7) and vimentin and suggested to be a crucial post-transcriptional regulator of hESC identity [71]. Beyond the five RNA-binding CSDs, CSDE1 was reported to contain four additional, interspersed CSDs that do not bind RNA [72]. In the guinea pig *UNR* gene, each CSD repeat is encoded by one exon, suggesting a modular assembly from one primordial gene [73].

Cold shock domain-containing protein C2 (CSDC2), also known as PIPPin (after a sequence motif inside its CSD), is a mammalian brain-specific protein that binds to histone mRNA and is thought to play a role in the regulation of brain development. CSDC2 was discovered as a protein that binds specifically to the 3′UTR of nuclear transcripts encoding rare histone variants. CSDC2 contains a central CSD preceded in the sequence by a presumably dsRNA-binding proline-rich PIP motif [74]. Along with E-cadherin, CSDC2 is induced by miR-373 and pre-miR-373 where the microRNA plays an unexpected role in transcription activation [75]. A further CSD-containing human protein, the calcium-regulated heat-shock protein 24 (CRHSP-24), was identified as a factor stabilizing the tumor-necrosis factor-α (TNF-α) mRNA by associating with its 3′UTR and thereby stimulating TNF-α production in a human cell line [76].

LIN28 is an essential RNA-binding protein that regulates the biogenesis of the let-7 family of tumor-suppressor microRNAs and modulates the translation of a large number of target mRNAs [52,77,78]. LIN28 also binds to the putative tumor-suppressor miRNA miR-363 [79]. Binding of LIN28 to microRNA precursors is mediated by an N-terminal CSD and a C-terminal zinc knuckle domain (ZKD) [80]. LIN28 mediates degradation of pre-let-7 by recruiting the 3′-terminal uridylyl transferase TUT4 to the microRNA [81] which thus becomes a substrate for the 3′-5′ exonuclease DIS3L2 [82]. The LIN28-mediated decrease in let-7 microRNAs causes overexpression of their oncogene targets including MYC, RAS, HMGA2 and BLIMP1 [83]. The LIN28—let-7 microRNA axis plays an important role in neuroblastoma development [84]. This is but one example for the broad involvement of LIN28 in human disease and particularly in cancer [85,86]. The LIN28—let-7 axis was also shown to be a central regulator of glucose metabolism by virtue of translationally repressing components of the insulin-PI3K-mTOR pathway [87]. The predominantly cytoplasmic human LIN28A and the nuclear LIN28B are the products of two closely related oncogenes. LIN28A can promote tissue repair by let-7-dependent as well as let-7-independent cellular mechanisms [88].

LIN28 has multiple let-7-independent cellular functions. Cross-linking immunoprecipitation with high-throughput sequencing (CLIP-seq) revealed GGAGA motifs as LIN28 binding sites within loops of approximately a quarter of all human transcripts. In somatic and pluripotent stem cells, LIN28 target sequences were found in mRNAs encoding LIN28 itself and splice regulators, suggesting functions in autoregulation and splicing [89,90].

Along with transcription factors OCT4, SOX2 and NANOG, LIN28 has been used to induce the reprogramming of human somatic cells into pluripotent stem cells [91]. The reprogramming ability of LIN28 is conserved in plants: The homologous cold-shock domain protein 1 (*Pp*CSP1) in the moss *Physcomitrella patens* regulates reprogramming of differentiated leaf cells into stem cells. *Pp*CSP1 is one of three paralogous *Pp*CSP proteins [92].

## 3. Structure of Cold-Shock Domains

The CSD is a simplified version of the OB fold lacking the α-helix. *B. subtilis* CspB was the first CSP to be crystallized [93], and its structure, determined from two crystal forms, set the paradigm for bacterial CSP and eukaryotic CSD conformation. The polypeptide chain is organized into an antiparallel five-stranded β-barrel with connecting loops of variable length. In the β-barrel, a three-stranded β-sheet and a two-stranded β-ladder are recognizable which are linked by only a few backbone hydrogen bonds. In spite of its small size of ~70 aa, the CSD contains a fully formed hydrophobic core. The presence of exposed aromatic residues on a basic protein surface strongly suggested a role in binding single-stranded nucleic acids, and ssDNA binding was confirmed in a gel-shift experiment [23]. The core findings of the crystallographic analysis of *Bs*CspB were essentially confirmed by the solution structure of the protein determined by nuclear magnetic resonance (NMR) spectroscopy [94]. Subsequently, a crystal structure of *Ec*CspA was determined at 2-Å resolution which revealed the same architecture as that of *Bs*CspB and a conserved nucleic acid-binding surface [95]. The solution NMR structure of *Ec*CspA was in general agreement with the crystallographic analysis and identified nine aromatic and two basic residues in binding to a 24-nucleotide ssDNA [96].

**Figure 3:**
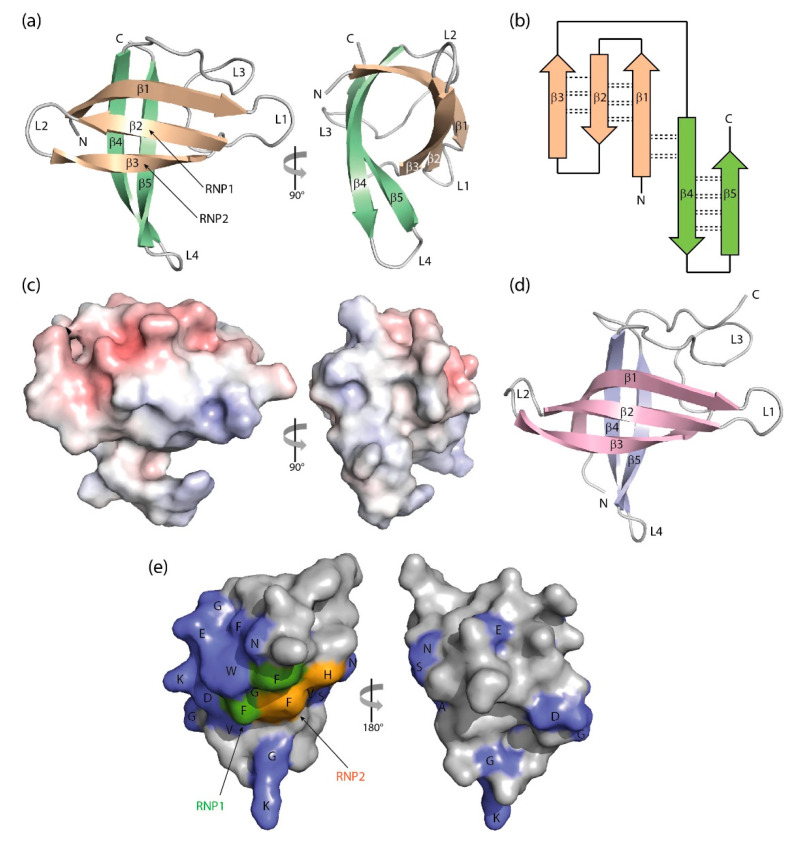
Three-dimensional structure of cold-shock proteins and domains determined at near-atomic resolution. (**a**) Cartoon drawing, (**b**) topology diagram with β-sheet stabilizing hydrogen bonds, and (**c**) electrostatic surface potential colored from red (−10 kT/e) to blue (+10 kT/e) of *Bc*CspB (PDB entry 1c9o). (**d**) Schematic drawing of the *X. tropicalis* LIN28 CSD (PDB entry 3ulj). Orthogonal views are presented in (**a**,**c**). (**e**) Conserved residues on the surface of *St*CspE (PDB entry 3i2z). RNP1/RNP2, RNA-binding motifs [20]. Note the close structural similarity between the bacterial *Bc*CspB [97] and the eukaryotic LIN28B CSD [100], the separation of negative (red) and positive (blue) surface charge in (**c**) and the asymmetric distribution of conserved residues over the CSP surface. Cartoon drawings were prepared with PyMOL [101], the topology diagram is based on PDBsum [102], and the electrostatic surface was calculated with the Adaptive Poisson-Boltzmann Solver (APBS) plugin [103] of PyMOL.

The crystal structure of *Bc*CspB at atomic resolution of 1.17 Å (Figure 3a,b) confirms the expected close conformational similarity with *Bs*CspB and suggests that the surface charge distribution (Figure 3c) may be linked to the enhanced thermal stability of this protein from the thermophilic *Bacillus caldolyticus* [97]. The NMR structure of the homologous *Tm*Csp from the hyperthermophilic *Thermotoga maritima* suggests residues mediating enhanced thermal stability in this CSP [98]. The structure of a psychrophilic CSP from *Listeria monocytogenes* determined by NMR resembles other bacterial CSP structures, but with a melting transition at 40 °C *Lm*CspA has reduced thermostability [99]. A variation in the structures of bacterial CSPs is offered by the NMR analysis of the single CSP from *Rickettsia rickettsii*, which shows a canonical CSP structure with the insertion of a short segment of α-helix in the long loop L3 [90].

In addition to bacterial CSPs, a number of ligand-free eukaryotic CSDs were structurally analyzed. The NMR structure of YBX1 CSD was the first structure of a eukaryotic CSD and proved that crucial structural features of the CSD are conserved from bacteria to man [104]. NMR analyses of all five canonical CSDE1 CSDs were also reported. These structures provide evidence for a special arrangement of several aromatic sidechains in the RNP motifs of CSD1 that differs from the other four CSDs and may be linked to the enhanced RNA-binding affinity of CSD1 [105]. The crystal structure of human CRHSP-24 reveals a canonical CSD preceded in the sequence by an α-helix. RNA binding by CRHSP-24 is regulated by phosphorylation at S41 [106]. The NMR structure of the single CSD of *Chlamydomonas reinhardtii* nucleic acid-binding protein 1 (NAB1), showing close similarity with *Bs*CspB and eukaryotic CSDs, was the first CSD structure from plants or green algae [107].

The single CSD and a tandem ZKD of LIN28 are both involved in nucleic-acid binding. The *Xenopus tropicalis* LIN28 CSD was shown to have a closely similar structure as bacterial CSPs (Figure 3d). The insertion of seven additional residues in loop L2 between β2 and β3 is accommodated by extending each strand by two residues and an altered loop structure [100].

The eukaryotic CSDs share with the homologous bacterial CSPs a markedly asymmetric surface distribution of conserved amino-acid sidechains (Figure 3e). Together with most conserved residues, the RNP1 and RNP2 motifs are located on one side of the CSD barrel, while just a few conserved sidechains are exposed on the backside of *St*CspE from *Salmonella typhimurium* [108], a protein containing all residues of the CSD consensus sequence according to Figure 1. The highly conserved residues include a set of exposed aromatic sidechains (marked W, F and H in the left panel of Figure 3e) playing a central role in DNA or RNA binding as detailed below.

### CSD β-Barrel Stability and Formation of Domain-Swapped Dimers

As a rule, bacterial CSPs and eukaryotic CSDs are monomeric under standard buffer conditions. However, the early crystal structure of *Bs*CspB [23] already provided evidence for weak dimerization of the protein which found support in a study by differential scanning calorimetry and size-exclusion chromatography suggesting that *Bs*CspB may be dimeric in the absence of phosphate [109]. The dimeric structure of two homologous CSPs from the psychrophilic *Bacillus cereus* was deduced from biochemical experiments [110]. Several more bacterial CSPs were described to form dimers whose spatial arrangement, however, remained undefined [111,112].

**Figure 4:**
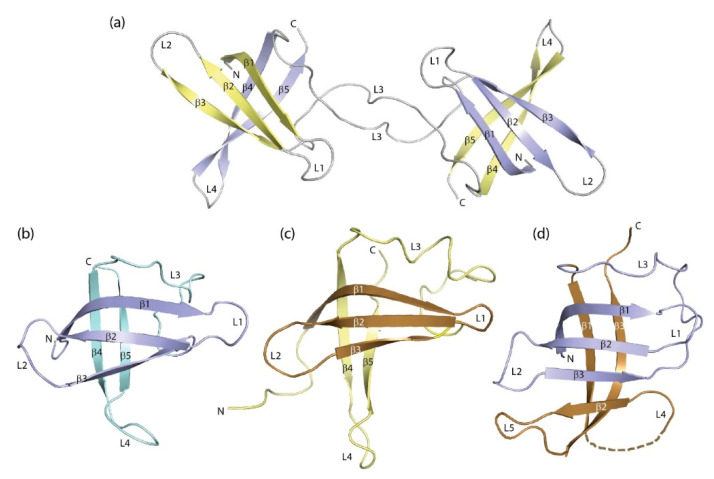
Domain- and segment-swapped forms of the cold-shock protein. (**a**) *Bc*CspB domain-swapped dimer ([113], PDB entry 2hax). Protein-bound DNA strands were omitted for the sake of clarity. (**b**) Crystal structure of *Ec*CspA ([95], PDB entry 1mjc) and (**c**) NMR structure of the S1 domain of *E. coli* polynucleotide phosphorylase ([117], PDB entry 1sro). Strands β1-β3 of both proteins that recombine to form 1b11 are highlighted by darker colors. (**d**) Structure of combinatorial protein 1b11 ([118], PDB entry 2bh8). Drawings prepared with PyMOL [101].

The crystal structure of DNA-bound *Bc*CspB revealed an unexpected domain-swapped dimer in which β-barrels closely resembling the commonly observed monomeric structures are formed by β-strands 1–3 from one subunit and 4 and 5 from the second (Figure 4a). Evidently, the formation of this dimeric structure was facilitated by the weak link connecting strands β3 and β4 in the barrel of the monomeric CSP (see Figure 3b), the rapid unfolding and refolding of bacterial CSPs (see below) and the high protein concentration used to grow crystals. The conformational change leading to dimerization is strictly limited to a single torsion angle in the peptide link between residues E36 and G37 of *Bc*CspB [113]. Deletion of the two residues at the hinge in the variant *Bc*CspBΔ36–37 leads to formation of a non-swapped protein dimer with a dimerization interface overlapping with the DNA/RNA-binding surface [114]. A domain-swapped dimer with very similar geometry as in *Bc*CspB was observed in the CSP from *Neisseria meningitidis* [115]. In principle, formation of domain-swapped dimers is possible with all proteins with unconstrained polypeptide chain termini, irrespective of secondary structure [116]. Dimer formation is favored under conditions of high protein density such as present during protein crystallization and in many cellular compartments. We are not aware of any domain-swapped dimers involving eukaryotic CSDs. If this type of self-association occurred with eukaryotic CSDs, it could have profound functional consequences.

In eukaryotic CSDs, an exon boundary frequently separates the N-terminal strands β1-β3 from the rest of the domain, suggesting that the CSD may have evolved by combination of the two elements. This observation prompted a study in which β1-β3 of *Ec*CspA (Figure 4b) were recombined at random with fragments of natural proteins. The crystal structure of one resultant protein, 1b11, in which three strands from *Ec*CspA have recombined with three strands from the S1 domain of *E. coli* polynucleotide phosphorylase (Figure 4c) shows a six-stranded β-barrel (Figure 4d) which represents one half of a domain-swapped dimer [118]. This structure illustrates the structural plasticity of the CSD and the related S1 domain in an impressive way.

## 4. Biophysical Properties of Cold-Shock Proteins

The presence in CSPs of a fully formed hydrophobic core and the absence of disulfide bonds or *cis*-peptides, often associated with slow phases in protein folding, rendered these proteins preferred targets for in-depth studies of their conformational stability, their folding kinetics and mechanism. With a free enthalpy of urea-induced unfolding at 25 °C of ΔG_D_(H_2_O) = 12.4 kJ mol^−1^ *Bs*CspB is only marginally stable, but it folds extremely fast in a reversible two-state reaction without folding intermediates. Urea-induced unfolding of *Bs*CspB proceeds with a time constant t_1/2_ = 20 ms, and refolding is characterized by a time constant t_1/2_ ≤ 1.2 ms [119]. *Bc*CspB from a thermophile and *Tm*Csp from a hyperthermophilic organism have significantly enhanced conformational stability, but retain the very fast two-state folding reaction [120].

Mutational analyses of *Bacillus* CSPs provided strong evidence that the surface charge distribution contributes strongly to conformational stability. Charge reversal of a single surface-exposed residue from arginine to glutamate accounted for two thirds of the stability difference between *Bc*CspB and *Bs*CspB [121] leading to the hypothesis that the removal of unfavorable Coulomb interactions on the surface of CSDs may be an optimal strategy for engineering conformational stability. The crystallographic analysis of five variants of *Bc*CspB carrying mutations of charged surface residues identified an acidic surface patch near the C-terminus that contributes to protein stability [122]. Molecular dynamics (MD) simulations strongly suggest that grafting additional favorable Coulomb interactions onto the surface of *Bc*CspB by directed mutagenesis may further enhance the CSP’s thermostability [123]. A similar observation was made when grafting stabilizing charge interactions from the surface of *Tm*Csp onto *Bs*CspB and studying the mutant CSP with single-molecule force spectroscopy (SMFS) and MD [124]. A full thermodynamic analysis of amino-acid contributions to the stabilization of a thermophilic CSP [125] that aimed at generating highly thermostable CSPs by in vitro selection yielded a *Bs*CspB variant with altered surface charges, an increase of the midpoint of the thermal transition by ~30 °C and of the Gibbs free energy of unfolding by ~21 kJ/mol [126,127]. In an alternative study, computational redesign of *Bs*CspB gave rise to a thermotolerant variant, CspB-TB, with increased transition temperature by ~20 °C [128]. A systematic study of engineered sequence variants of CspB-TB led to the conclusion that charge-charge interactions on the surface of the folded protein (and not in the unfolded state) are mainly responsible for the observed structural stabilization [129].

CSPs were characterized as two-state folders in ensemble experiments [119,120]. Recently, however, SMFS of *Tm*CspB revealed multiple long-lived unfolding intermediates [130]. The apparent discrepancy between unfolding experiments in bulk and with single molecules was reconciled by coarse-grained MD simulations demonstrating that *Tm*CspB unfolding intermediates can be stabilized by the pulling force [131].

## 5. Cold-Shock Domain-Binding to Nucleic Acids

In spite of the small size and simple architecture of CSPs and CSDs, their DNA- or RNA-bound three-dimensional structures are still limited in number, and many were published only recently. The following compilation will show that both bacterial CSPs and eukaryotic CSDs (i) bind their single-stranded nucleic-acid ligands with closely similar geometry, (ii) do not discriminate much between ssDNA and ssRNA binding, and (iii) display limited DNA or RNA sequence selectivity.

### 5.1. Bacterial Cold-Shock Proteins

#### 5.1.1. DNA Binding

**Figure 5:**
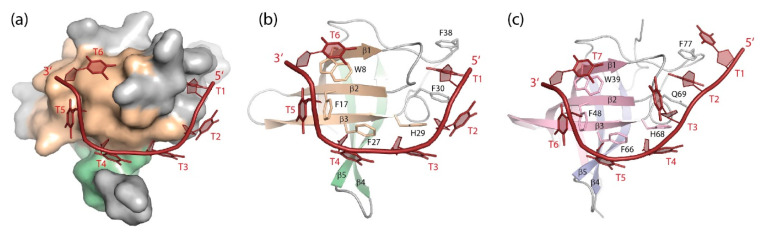
DNA single strands bound to CSDs. (**a**) Solvent-accessible surface and (**b**) cartoon drawing of *Bc*CspB bound to (dT)_6_ ([113], PDB entry 2hax). Only one globular unit formed by two strands of a domain-swapped *Bc*CspB dimer (see Figure 4) is displayed. (**c**) (dT)_7_ bound to LIN28B CSD ([100], PDB entry 4a76). Note how stacking interactions between nucleobases and aromatic amino-acid sidechains contribute prominently to the closely similar binding interfaces of the bacterial CSP and the eukaryotic CSD. Drawings prepared with PyMOL [101].

Probing the DNA-sequence selectivity of *Bs*CspB with a DNA microarray-based approach revealed the pyrimidine-rich heptameric consensus sequence d(GTCTTTG/C) [132], and an *in vitro* analysis confirmed that binding affinity of DNA fragments to *Bs*CspB correlated with thymidine content [133]. Following these leads, *Bs*CspB and *Bc*CspB were both crystallized in complex with the single-stranded DNA fragment (dT)_6_ [134]. The crystal structure of (dT)_6_-bound *Bs*CspB (Table 1) revealed the general principles of oligonucleotide binding to CSPs or CSDs, although the *Bs*CspB-bound DNA strand was discontinuous in this particular structure. A DNA single strand binds in a groove across a positively charged protein surface with exposed aromatic sidechains. The DNA or RNA bases are oriented towards the protein, stacking atop aromatic protein sidechains and forming a limited number of hydrogen-bonded interactions with the protein backbone or sidechains, whereas the DNA or RNA backbone faces the solvent and is not in contact with the protein [135]. This binding geometry offers an explanation why CSPs display limited sequence specificity and discriminate poorly between DNA and RNA strands. NMR and mutational analyses identified a similar set of ssRNA-binding sites in *Bs*CspB [136].

(dT)_6_ binds to the homologous *Bc*CspB with similar geometry as to *Bs*CspB, but with a contiguous DNA strand (Figure 5a,b). The six base-binding subsites are conserved between both proteins [113] and formed by highly conserved protein sidechains including those in the RNP1 and RNP2 motifs (see Figure 1). DNA and RNA oligonucleotide binding to *Tm*Csp was mapped by NMR chemical-shift perturbations revealing nucleic acid-protein contacts as observed in other bacterial CSPs, although the full structure of a *Tm*Csp:oligo(deoxy)ribonucleotide complex was not determined [138].

#### 5.1.2. RNA Binding

**Figure 6:**
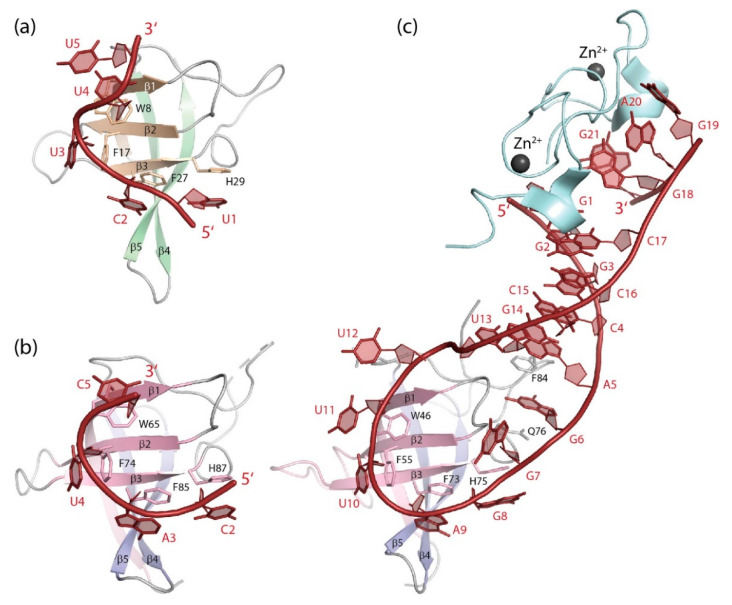
RNA single strands bound to CSDs. (**a**) GUCUUUA bound to *Bs*CspB ([139], PDB entry 3pf4). The 5′ and 3′-terminal nucleotides of the co-crystallized RNA strand are not revealed in the structure. (**b**) UCAUCU bound to the YBX1 CSD ([11], PDB entry 5ytv). The 5′ and 3′-terminal nucleotides of the co-crystallized RNA strand are not revealed in the structure. (**c**) The microRNA precursor preE_M_-let-7d bound to the CSD and ZKD of mouse LIN28A ([140], PDB entry 3trz). Drawings prepared with PyMOL [101].

Pyrimidine-rich ssRNA fragments bind *Bs*CspB (Table 2) with closely similar geometry as their DNA analogs, and their bases occupy some of the same subsites on the cold-shock protein surface (Figure 6a). DNA strands, however, bind *Bs*CspB with ~10-fold higher affinity than analogous RNA strands. This difference in binding strength is shown to arise from favorable contributions to the binding energy of the thymine methyl groups and is not provided by the nucleic-acid backbone [139].

### 5.2. Eukaryotic Cold-Shock Domains

#### 5.2.1. DNA Binding

Analyses by NMR and isothermal titration calorimetry (ITC) of DNA-heptamer binding to an extended YBX1 CSD reveals DNA-sequence preferences of YBX1, a binding mode resembling bacterial CSPs and the structural basis of the observed attenuated target DNA binding by S102 phosphorylation [9]. Conversely, dephosphorylation of S102 and other serine residues was proposed to unmask the nuclear localization signal of YBX1 and facilitate nuclear entry at specific stages during the cell cycle [141]. Protein kinase AKT-mediated phosphorylation of YBX1 also regulates its binding to the capped 5′-end of mRNA [142]. Whereas the phosphorylation site at S102 is located in loop L3 within the CSD, a recently described site of O-glycosylation at T126 [143] is just outside the CSD and not revealed in any structural study. This threonine glycosylation was shown to affect S102 phosphorylation, thereby enhancing cell proliferation in hepatocellular carcinoma [143]. Biological effects arising from ssDNA binding by YBX1 have been widely reported. For example, YBX1 binds ssDNA in the MHC class-II DRA promoter resulting in transcriptional repression [144], and YXB1 binds to an enhancer sequence in the *PTP1B* promoter regulating the cellular levels of this protein tyrosine phosphorylase [145].

(dT)_6_ and (dT)_7_ bind to the LIN28B CSD with similar geometry as to bacterial CSPs regarding strand polarity and base-binding subsites [100]. Subsite occupation by DNA bases is nearly identical in *Bc*CspB and LIN28B CSD with one additional subsite in the latter being occupied by the 5′-terminal thymidine (Figure 5c). To date, most cellular functions of LIN28 have been linked to RNA binding. However, it was reported that LIN28A binds a consensus DNA sequence both in vitro and in mouse embryonic stem cells. By recruiting the 5-methylcytosine dioxygenase TET1 to specific genomic sites, LIN28A assumes a previously unexpected role as epigenetic and transcriptional regulator [146,147].

#### 5.2.2. RNA Binding

High-resolution crystal structures of YBX1 bound to four different RNA-hexamer strands (Figure 6b) reveal a binding geometry in which the bases of the central CAUC core motif or variants thereof are stacked onto four conserved aromatic protein sidechains [11]. CAUC and CACC motifs had previously been identified by systematic evolution of ligands by exponential enrichment (SELEX) as high-affinity YBX1-binding sites. This study suggested a role of YBX1 in mRNA splicing by recruitment of splicing factors to certain splice sites [148]. Very recently, YBX1 was identified as a JAK2 protein-kinase target whose inactivation caused intron retention in proteins of the ERK signaling pathway [149]. UCCAUCA was also identified as target sequence for mouse YBX2 and YBX3 in the 3’UTR of the *PRM1* (protamine 1) mRNA [150]. Human YBX3 binds a similar set of mRNAs as its homolog YBX1 [151], suggesting that target selection is driven by the CSD which has identical sequence in YBX1 and YBX3, but not by the flanking regions which differ between both proteins (see Figure 1 and Figure 2).

In human bladder-cancer cells, the NOP2/Sun RNA methyltransferase 2 (NSUN2) methylates cytosine bases. YBX1 binding to a 5-methylcytosine (m^5^C) site within the 3′UTR of the oncogene mRNA of heparin binding growth factor (HDGF) stabilizes this mRNA, thereby contributing to oncogene activation. The crystal structure analysis shows that YBX1 recognizes the m^5^C-modified target mRNA through interaction with its W45 sidechain [12]. Residue W45 in zebrafish YBX1 is essential for the preferential recognition of m^5^C-containing RNA as well, as shown by crystal structure analysis [11]. This interaction is crucial for maternal mRNA stabilization in the maternal-to-zygotic transition in early zebrafish embryogenesis [10]. Finally, the *Drosophila* YBX1 homolog Ypsilon schachtel (YPS) binds preferentially to m^5^C-containing RNA, thereby promoting stem-cell maintenance, proliferation and differentiation. As YBX1, YPS binds m^5^C-containing oligoribonucleotides in vitro with higher affinity than unmodified RNA, a behavior reminiscent of the preferred binding of DNA over RNA strands to *Bc*CspB which was linked to the thymine methyl groups [139]. Crystal structure analysis shows that the ssRNA octamers ACCAGm^5^CCU and ACCAGCCU bind the YPS CSD with similar geometry with both m^5^C6 and C6 binding to the same subsite by stacking onto the sidechain of residue F85 [8]. In all studied CSDs, m^5^C-containing RNA strands were bound with closely similar geometry as the unsubstituted RNAs.

On a higher structural level, a combination of small-angle X-ray scattering (SAXS), NMR and MD simulations [11] was used to characterize filaments of an mRNA-bound C-terminally truncated YBX1. This work leads to the hypothesis that YBX1 may have a role in unfolding structured mRNA molecules [152].

Gene dosage compensation between female and male flies is mediated by the *Drosophila* gene *male specific lethal-2* (*msl2*). The translation of *msl2* mRNA is down-regulated by the proteins Sex-lethal (SXL) and CSDE1 that both bind in the *msl2* 3′UTR. A crystal structure of a ternary complex formed by SXL, the CSDE1 CSD1 and an 18-nucleotide RNA from the 3′-region of *msl2* mRNA [14] demonstrates that this CSD follows the paradigm established before for homologous domains in its mode of RNA binding. To our knowledge, this is so far the only structure showing details of the cooperative binding of a CSD and another RBP on the same RNA strand.

A systematic study of LIN28 binding to the human transcriptome confirmed the predicted binding to precursors of let-7 microRNAs and revealed association with the majority of mRNAs, both in their coding sequences and 3′UTRs [153]. Both in vitro and in vivo LIN28 proteins preferentially bind to uridine-rich single-stranded RNA [154].

Crystallographic analysis revealed fine structural details of mouse LIN28A binding to three different let-7 microRNA precursor elements: preE_M_-let-7d (Figure 6c), preE_M_-let-7f-1, and preE_M_-let-7g. The structures reveal that the LIN28A CSD has the ability to accommodate rather different stem-loop structures while the ZKD binds a GGAG motif in all pre-micro-RNAs, and the truncated linker peptide remains unresolved in any structure [140]. Biochemical evidence suggests that LIN28-CSD binding to the terminal loop structure of pre-let-7 microRNA precedes and facilitates binding of the ZKD to a conserved GGAG motif in the microRNA precursor [100]. A study of the binding mechanism of LIN28 with the let-7g terminal loop supports the notion of a stepwise protein binding with RNA remodeling by the LIN28 CSD [155]. MD simulations of the LIN28 interaction with different microRNA subtypes lends further support to a sequential binding of the CSD and ZKD to pre-let-7 [156].

There is evidence for the existence of two subclasses of let-7 microRNA precursors, one with binding sites for the LIN28 CSD and ZKD and one with only ZKD binding sites [157] and that the specific association of the LIN28 ZKD with pre-let-7 is required and sufficient for recruitment of the terminal uridylyltransferase TUT4 [13]. For pre-let-7g, biochemical data suggest that a basic region in between the LIN28 CSD and ZKD contributes to binding [158]. Finally, it may be noted that small molecules were identified by screening a compound library that inhibit both LIN28:let-7 binding and LIN28-mediated RNA polyuridylation, thus opening an avenue towards pharmacological intervention with the oncogenic activities of LIN28. One inhibitor, TPEN, is directed against the LIN28 ZKD, whereas a second inhibitor, LIF1, was shown to bind to the LIN28 CSD and prevent its RNA binding [159].

## 6. Conclusions

In this review, it was attempted to provide a structural basis to explain how an evolutionarily conserved simple protein module, the CSD, is able to support a wide variety of biological functions ranging from transcriptional regulation and DNA repair to the control of RNA splicing, stability, translation and sequestration. The wide-ranging similarity between bacterial CSPs and eukaryotic CSDs regarding their sequences, three-dimensional structure and nucleic-acid binding is clearly revealed. CSD-containing proteins from all kingdoms of life share a common mode of nucleic-acid binding which is characterized by stacking between nucleobases and aromatic protein sidechains, a solvent-exposed sugar-phosphate backbone and conserved strand polarity, where the 5′-end of the bound DNA or RNA single strand is near the C-terminus of strand β1 and the 3′-end is in the vicinity of the C-terminus of β2 on the CSD β-barrel. In addition, all CSDs share a conserved set of nucleotide-binding subsites involving the most highly conserved residues across the large number of CSD sequences. This binding geometry readily explains the limited discrimination of single-stranded DNA from RNA by CSDs and their lack of distinct sequence specificity. The participation of CSDs in a wide range of cellular functions may be understood as a direct consequence of this common nucleic acid-binding mode.

The biochemical, biophysical and structural properties of CSDs reviewed here are the basis for all biological functions of CSD-containing proteins. However, they are insufficient to fully understand the functions of YBX1, LIN28 and other human proteins in a broader, e.g., cancer context, because these functions also depend on the natively unfolded parts of these polypeptides, their tissue distribution, sub-cellular localization and expression levels. In this context, it may be recalled that the human and murine YBX1, YBX2 and YBX3 proteins have very closely matching CSD sequences, but clearly different mutant phenotypes [22]. Open questions regarding the molecular basis of the unique association of YBX3 with the tight-junction protein ZO-1 [64,65], the propensity of YBX1 to alternatively stimulate or repress translation of target mRNAs [160] or the coordination of the various roles of YBX1 in disease [53,54,55,56] are at least partly linked to the natively unstructured regions of these proteins, and their resolution will require further research.

Some aspects of CSD stability, folding and structural plasticity have been much more thoroughly studied in bacterial CSPs than in eukaryotic proteins. Given the conservation of sequence, structure and nucleic-acid binding across all members of the large CSD family, it is suggested that for understanding human CSDs much may still be learnt from bacterial CSPs, especially regarding conforma-tional stability and structural plasticity.

## Figures and Tables

**Table 1:** Structures of CSP/CSD: DNA complexes.

CSP/CSD	Organism	Sequence ^1^	Method	PDB ID ^2^	Reference
*Bs*CspB	*B. subtilis*	TTT TTT	X-ray	2es2	[135]
*Bc*CspB	*B. caldolyticus*	TTT TTT	X-ray	2hax	[113]
LIN28B CSD	*X. tropicalis*	TTT TTT TTT TTT T	X-ray X-ray	4a75 4a76	[100]
YBX1 CSDex ^3^	human	AAC ACC T	NMR	6lmr	[9]

^1^ Residues in contact with the CSP/CSD are underlined. Other nucleotides may be disordered or not observed. ^2^ Entry in the Protein Data Bank [137]. ^3^ C-terminally extended CSD covering residues D51-A140.

**Table 2:** Structures of CSP/CSD: RNA complexes.

CSP/CSD	Organism	Sequence ^1^	Method	PDB ID ^2^	Reference
*Bs*CspB	*B. subtilis*	UUU UUU GUC UUU A	X-ray	3pf5 3pf4	[139]
LIN28A CSD+ZKD	*M. musculus*	GGG CAG AGA UUU UGC CCG GAG ^3^ GGG GUA GUG AUU UUA CCC UGG AG ^4^ GGG GUC UAU GAU ACC ACC CCG GAG ^5^	X-ray	3trz 3ts0 3ts2	[140]
LIN28B CSD	*X. tropicalis*	UUU UUU	X-ray	4alp	[100]
CSDE1 CSD1 ^6^	*D. melanogaster*	UUU UUU UGA GCA CGU GAA	X-ray	4qqb	[14]
LIN28A CSD+ZKD	human	GGG GUA GUG AUU UUA CCC UGG AGA U	X-ray	5udz	[13]
YBX1 CSD	human	UCA UCU UCU UCU UCA ACU UCA UGU	X-ray	5ytv 5yts 5ytx 5ytt	[11]
YBX1 CSD	human	UCA Um ^5^ CU	X-ray	6a6l	[12]
YBX1 CSD	*D. rerio*	UCA Um ^5^ CU	X-ray	6a6j	[10]
YPS CSD	*D. melanogaster*	ACC AGC CU ACC AGm ^5^ C CU	X-ray	6kug 6ktc	[8]

^1^ Residues in contact with the CSP/CSD are underlined. Other nucleotides may be disordered, not observed or contacting other structural elements of the protein. ^2^ Entry in the Protein Data Bank [137]. ^3^ preE_M_-let-7d; ^4^ preE_M_-let-7f-1; ^5^ preE_M_-let-7g. ^6^ In ternary complex with a protein fragment containing domains RRM1 and RRM2 of Sex-lethal (SXL).
